# Acknowledgment to Reviewers of *Viruses* in 2021

**DOI:** 10.3390/v14020251

**Published:** 2022-01-27

**Authors:** Viruses Editorial Office

**Affiliations:** MDPI AG, St. Alban-Anlage 66, 4052 Basel, Switzerland

Rigorous peer-reviews are the basis of high-quality academic publishing. Thanks to the great efforts of our reviewers, *Viruses* was able to maintain its standards for the high quality of its published papers. Thanks to the contribution of our reviewers, in 2021, the median time to first decision was 17 days and the median time to publication was 38 days. The editors would like to extend their gratitude and recognition to the following reviewers for their precious time and dedication, regardless of whether the papers they reviewed were finally published:
Aa Haeruman AzamJudd F. HultquistAarthi NarayananJudit J. PénzesAbdul A. WaheedJudith BoumanAbdullah ElyJudith DavieAbdullah Sami SaribaşJudith OlejnikAbhijeet BakreJudith Van Den BrandAbhishek ShrivastavaJudith WhiteAbimbola KolawoleJudy MitchellAbimbola O. KolawoleJuergen RichtAbraham PouliakisJukka T. MustonenAchim QuaiserJulia AlmeidaAchim TemmeJulia Béjar AlvaradoAd De GroofJulià BlancoAd VosJulia DinaÁdám BálintJulia GrasslAdam GlowaczJulia KenyonAdam LiwoJulia R. ClarkeAdam ObersteinJulia ShackelfordAdam StewartJulian Ruiz-SaenzAdam W. WhisnantJulián Ruiz-SáenzAdarsh K. GuptaJuliana G MelgaçoAdel DrissJuliana Reis CortinesAdhikarimayum Lakhikumar SharmaJulie FoxAdi BeharJulien AndréaniAdly Mohamed M. Abd-AllaJulien ClaudeAdolfo PomaJulien PomponAdrian Alejandro ValliJulien Van GrevenyngheAdrian Von WitzlebenJuliet MorrisonAdriana DelfraroJuliet V. SpencerAdriana HeguyJulio J. DiezAdrianne JennerJumpei FujikiAdriano De Bernardi SchneiderJumpei ItoAdrien BreimanJun AriiAgam RaoJun HangAgieshkumar Balakrishna PillaiJun InoueAgnes KocherJun KuritaAgnieszka A. KaczorJung-Eun ParkAgnieszka Olejnik-SchmidtJunhua LiAhmed AliJunichi TakinoAhmed ElaswadJunki MaruyamaAhmed ElbestawyJunsoo ParkAhmed KheimarJürg NüeschAhmed MostafaJürgen GebelAileen Y. ChangJürgen RödelAisha NazliJürgen WenzelAitor NogalesJustin A. RobyAjit MagadumJutta PikaloAke LundkvistK. Scott WeberAkihide RyoKai Alexander KroppAkihiro SugawaraKai KappertAkiko KashiwagiKai LingAkira NishizonoKaining ZhiAkira OnoKaishu LingAlain VanderplasschenKaján GyőzőAlan CochraneKamalendra SinghAlan G. GoodmanKang Hao CheongAlan L. SchmaljohnKannan GanapathyAlan ParkerKaori TerasakiAlan ReinKaoru TakeuchiAlba Cecilia Ruiz-GaitánKapil GuptaAlban RametteKappei KobayashiAlbert BoschKaren BeemonAlbert NienhausKaren KirbyAlbert ZimmermannKaren KormuthAlberta AzziKaren V. KiblerAlberto A. AmarillaKaren WeynbergAlberto Enrico MaraoloKarin DarpelAlbie Van DijkKarin E. KlingelAlbino CarrizzoKarl RobinsonAldo MarroneKarla PassalacquaAlec GerryKarsten TischerAlejandra Gutierrez-GuerreroKashif Aziz KhanAlejandro BandaKatarina TrajkovićAlejandro Marin LopezKatarzyna Domańska-BlicharzAlejandro ValbuenaKatarzyna OtulakAleksandra KalitnikKatarzyna PodgórskaAleksandra PawlakKatarzyna Szot-KarpińskaAleksandra Petrovic FabijanKate WardAles KovarikKateri BertranAlessandra GaffuriKaterina PsarraAlessandra MozziKaterina RoubalovaAlessandra PierangeliKaterina StratiAlessandro LoglioKaterina TrejbalovaAlessandro MalobertiKaterina TsiokaAlessandro MangognaKatharina JandlAlessandro MarcelloKatharina L. LohmannAlessandro SinigagliaKatharine MagorAlessandro TavelliKatherine BrunerAlessia GiordanoKatherine Laiton-DonatoAlessio BocediKatherine SchultheisAlessio CantoreKatherine Seley-RadtkeAlex ComptonKatherine YoungAlex EvilevitchKathleen B. CartmellAlex M. MurphyKathleen Boris-LawrieAlex Pauvolid-CorrêaKathleen HefferonAlex RouvinskiKathleen Hooper-McGrevyAlexander Batista-DuharteKathleen L. HefferonAlexander BolshoyKathleen Laura HefferonAlexander DomnichKathleen McCaffreyAlexander HahnKathleen PappritzAlexander HynesKathlyn LavalAlexander J. McAuleyKathrin HeldAlexander KaraulovKathrin SpendierAlexander LaiKathryn KauffmanAlexander M. ShestopalovKatie TormanenAlexander MottKatja C. WolthersAlexander N. FreibergKatja FinkAlexander NovikovKatrien PoelaertAlexander PasternakKatrin Helfer-HungerbühlerAlexander PostelKatsuhiro KomaseAlexander S. MalogolovkinKatsuhito KinoAlexander SchultzeKatsumi MizutaAlexander TavellaKatsunori OkazakiAlexander V. ProninKaveesha WijesingheAlexander W. E. FranzKaw Bing ChuaAlexandra MalbonKayvan EtebariAlexandra SchaeferKazuki ShimizuAlexandre EscargueilKazumichi FujiokaAlexandre GaymardKazunori NagasakaAlexandre LeitãoKazuo NishigakiAlexandros PatrianakosKazushi MotomuraAlexey AgranovskyKazuya NaganoAlexey DeykinKeiji UedaAlexis BouinKeiko Kadono-OkudaAlfredo Diaz-LaraKeir M. BallaAlhammad YousefKeisuke NakagawaAli AbdallahKeita MatsunoAli Ait HssainKeith ChappellAli RostamiKeith LauAlice ChateauKeith R. JeromeAlice MichieKelli L. BarrAlice VassiliouKellie Ann JuradoAlicia K. OlivierKelly S. HarrisonAlicia Martínez-VareaKen LemonAlicia McLuckieKen RosenthalAlicia OteroKen SatoAlicja WegrzynKendal RyterAlicja WęgrzynKenji S. NakaharaAlise J. PonseroKenneth A. AndreoniAlison AshbrookKenneth A. StaplefordAlistair RussellKenneth CornettaAllan R. BrasierKenneth H. EckelsAllen HerbstKenneth LundstromAlok ChoudharyKenneth ReidAlteri ClaudiaKenneth StaplefordAlvaro MallagarayKensaku MaejimaAlvydas MalakauskasKenta ShimizuAlyson KelvinKentaro YamadaAmanda Carroll-PortilloKerstin MairAmar ParvateKerstin RosenbergerAmber JollyKetki TendulkarAmelia K. PintoKevan L HartshornAmelina AlbornozKevin CassadyAmina KhatunKevin CiminskiAmir SteinmanKevin CoombsAmirbabak Sioofy-KhojineKevin D. PavelkoAmir-Babak Sioofy-KhojineKevin FonsecaAmit KumarKevin LahmersAmol KulkarniKevin M. CoombsAmr GamilKevin MaringerAmulya YaparlaKevin RoedlAmy GilbertKevin SokoloskiAmy JacobsKevin TsaiAmy LambertKhalid MuhammadAmy Turmelle GilbertKhalid ShahinAna Belen Ruiz-GarciaKhalid TimaniAna Cecília Ribeiro CruzKi Hyun NamAna DomenechKi-Jong RheeAna KlobucarKim BlasdellAna Maria Rivas-EstillaKim GreenAna MartinezKim HalpinAna MorenoKim LeMessurierAna P. LunaKim SneppenAna ReisKim SteegenAna Rita FerreiraKimberly M ChristianAna-Belen BlazquezKimmo VirtanevaAnan JongkaewwattanaKinjal MajumderAnanda Ayyappan Jaguva VasudevanKinjiro MorimotoAnastasia HyrinaKiran TotiAnastasia KotanidouKiril DimitrovAnat ZviKirill SharshovAnbu Kumar KaruppannanKirill VasilyevAnca DoceaKirsten SpannAnders BoydKirsten WhiteAnders FomsgaardKiyotaka ShibaAnders NäsmanKlaus HamprechtAndi KrumbholzKlaus StrebelAndor DoszpolyKobporn BoonnakAndré Luis Costa-Da-SilvaKoen BartholomeeusenAndréa Alice Da SilvaKohji MoriishiAndrea CarfiKohtaro MiyazawaAndrea ChavesKonstantin MiroshnikovAndrea CimarelliKonstantin M.J. SparrerAndrea GalliKonstantinos StamatakisAndrea GiacomelliKoray ErgunayAndrea GiansantiKore SchlottauAndrea KressKornelia HardesAndrea KroekerKornélia KuruczAndrea MarcinnòKosuke OkuyaAndrea Moreno SwittKouichiro KawanoAndrea RaymondKow-Tong ChenAndrea Saul CostaKripitch SutummapornAndrea Thoma-KressKrishna Chaitanya SuddalaAndrea VögtlinKrishna Das ManandharAndreas BeinekeKrishnaswamy Gopalan TirumurugaanAndreas HillarpKristen OgdenAndreas KuhnKristin KöppenAndreas KurthKristin ParentAndreas SuhrbierKristina M. MillerAndreas WackKristine WylieAndree ZibertKrystyna Bieńkowska-SzewczykAndrei A. DeviatkinKrystyna Da̧browskaAndreia MóscaKrzystof TomasiewiczAndres Blanco CarriónKrzysztof SzczubiałkaAndrés Velasco-VillaKrzysztof SztankeAndrew D. MillardKrzysztof TrederAndrew HendersonKS BenschopAndrew HickeyKsenia EgorovaAndrew J. HendersonKuanhui XiangAndrew J. ThompsonKun HuangAndrew LangKun ZhangAndrew MillardKunihiko UmekitaAndrew NorrisKuo-Hu ChenAndrew ReadKurt GustinAndrew RiceKurt ToblerAndrew ShawKwai Fung HuiAndrew YurochkoKyle RosenkeAndrey A. FilippovKylene Kehn-HallAndrey A. GorchakovKylie M. WagstaffAndrey LetarovKym LowryAndrey SimbirtsevKyoko Tsukiyama-KoharaAndrey SolovyevKyriacos A. MitrophanousAndrzej Krzysztof SiwickiKyung H ChoiAndrzej LangeKyung Hyun KimAngel L. OrtegaKyung-Yil LeeAngela CastoldiLabrini V. AthanasiouAngela CrawleyLadislau KovariAngela M. Bosco-LauthLaetitia CaniniAngela SousaLaëtitia Trapp-FragnetAngela WitteLaila GasmiAngelica BiancoLaila Sara Arroyo MührAngelica StranieriLanlan BaiAngelo PavesiLara ManganaroAngelo SpenaLara Valiño-RivasAngus BucklingLarance RonsardAngus WilsonLarry A. HansonAnice LowenLarry DishawAnissa ChouikhaLászló EgyedAnita M. SheteLászló PalkovicsAnja Kathrin WegeLaura De PlanoAnja KiparLaura E. BrettellAnju BansalLaura GallinaAnke Renate Maria KraftLaura HertelAnke SchwarzLaura KalinieneAnn SheehyLaura KasmanAnna BaraniakLaura Medina-PucheAnna C. MaurerLaura N. BurgaAnna CamporesiLaura PellegrinelliAnna Chi Man TsangLaura PetrarcaAnna Halling Folkmar AndersenLaura RitcheyAnna JackovaLaura TomassoneAnna K. WalduckLaura ZaniAnna Karolina MatczukLaurel L. LenzAnna KlocLauren A. SiltzAnna KolliopoulouLauren M. SmithAnna KoromyslovaLaurence AlbarAnna LacastaLaurent AndreolettiAnna Linda ZignegoLaurent Chatel-ChaixAnna LopataLaurent DacheuxAnna Louise FunkLaurent GilletAnna LuganiniLaurent MullerAnna Maria SardanelliLaurie KrugAnna Maria VairaLauro Velazquez-SalinasAnna OrłowskaLavinia FabeniAnna PikulaLavinia Schuler FacciniAnna PoimalaLea HošnjakAnna Rosa GarbugliaLéa LucianiAnna S. FahrionLee Lee TengAnna SalvettiLee MakowskiAnna TaglientiLee SherryAnna WaldLeena MaunulaAnnalisa ChiavaroliLeena ShewadeAnne DalmonLeendert A. TrouwAnne HaleniusLeila GironAnne Harduin-LepersLemonia SkouraAnne PohlmannLen SeymourAnne SimonLena AllweissAnne WyllieLeó PomarAnnekathrin HaberlandLeona GilbertAnnelies KeymeulenLeonardo SustaAnne-Sophie Van LaereLeonid ChindelevitchAnnette AudigéLeonidas LotosAnnette MartinLeor S. WeinbergerAnnika BrinkmannLes DomierAnshuman DasLeslie Chavez-GalanAnthony MarriottLeslie D. SimsAntoine TouzéLeslie ParentAntoinette Van Der KuylLester M. ShulmanAnton SholukhLeszek TylickiAntonella CaputoLeticia V. BentancorAntonella Di SottoLi HanAntonella FrassanitoLi YinAntonella MinutoloLia Van Der HoekAntonella VirgilioLiam P. ShawAntonia P. SagonaLianbing LinAntonio ArtiguesLidia Szulc-DąbrowskaAntonio C.P. WongLihua WangAntonio FernándezLi-Kwan ChangAntonio González-BulnesLili HanAntonio Gregorio Dias JuniorLili YangAntonio J. Martin-GalianoLiliang JinAntonio José Piantino FerreiraLily Hui-Ching WangAntonio MasLinda DixonAntonio MoriLinda ScobieAntonio Rivero-JuárezLing JinAntonio RussoLionel BerthouxAntonios KatsounasLionel FrangeulAnuj AhujaLionel HebbardApostolos BeloukasLisa G. NevenAppavu SundaramLi-Teh LiuAraceli CastilloLiubov KozlovskayaArcangeli GiuseppeLivia DonaireArianna CalistriLivia PatronoAriful IslamLivia StavoloneArijita SarkarLizabeth AllisonArinze OkoliLizhou ZhangAris T. SpathisLjubo BarbicArmando Andres Roca SuarezLlilianne GangesArmando AriasLogan BanadygaArmen GasparyanLoic LegrantArmin EnsserLokesh SharmaArnold GrünwellerLone BrondstedArnon DishonLong-Ding LiuArrigo CiceroLorenz LeitnerArsalan S. HaqqaniLorenz Lorenz RischArtem G. LadaLorenzo FraileArtem MetlinLorenzo OnoratoArtem VorobyevLorne BabiukArtemis RumbouLouis ShekhtmanArthur GruberLouise CosbyArthur KocherLouise PittArthur R. FramptonLuan VuArun K. DharLuca Danilo BertzbachArun Kumar TharkeshwarLuca De SabatoAruna AmbagalaLuca FalzoneArvind VarsaniLuca MarchettiAshish GoyalLuca NervaAshok BhagwatLuca PeruzzaAshwanth Christopher FrancisLucía FernándezAsit KumarLuciana CostaAsit PattnaikLuciano Pamplona De Góes CavalcantiAspen M. WorkmanLuciano PironeAsrar AhmadLucio GamaAsuka Hirai-YukiLudovic MartinelleAtanu K. KhatuaLuigi SantacroceAthanassios K. GiatropoulosLuigi VetrugnoAthar AnsariLuigi VimercatiAthe TsibrisLuis Antonio Mariscal-AmaroAtsushi HIjikataLuis Del ValleAtsushi KatoLuis F. NuñezAtsushi YamanakaLuis G.C. PachecoAttila TóthLuis Gonzalez De PazAudray K. HarrisLuis Menéndez-AriasAudrey EsclatineLuis OrtegaAudrey LacroixLuis Perez Garcia-EstañAugustin Mouinga-OndéméLuisa AccardiAugustine GubbaLuísa PereiraAura R. GarrisonLuka Cicin-SainAurélie VelayLuka MesinAusra DomanskaLukas HegerAvinash WaghrayLukas LacinaAyato TakadaLukasz KurykAymeric HansŁukasz ŚwiątekAyumi SugiuraLuke R IwanowiczAyushi RaiLung-Yi MakAzumi IshizakiLutz GöhringBaki AkgülLuwanika MleraBalik DzhambazovLuz Martín-CarboneroBandar AlosaimiLydia TangBangxing HongLyn WiseBarbara BakerLynnette C. GoatleyBarbara BiereM. D. SalmanBarbara KluppM. Del Camino De Juan RomeroBarbara MaciejewskaM. R. BrunettoBarbara MüllerMa Cristina Del Rincón-CastroBarbara RuaroMaarit SuomalainenBarbara S. DroletMaciag-Dorszyńska MonikaBarbara SchmidtMaciej ZielińskiBarry MilavetzMadalena Vieira-PintoBarry R. PalmerMagaly MartinezBarry RockxMagdalena BaymakovaBartłomiej GrygorcewiczMagdalena DunowskaBartosz ForoncewiczMagdalena FiglerowiczBasavaprabhu PatilMagdalena PłotkaBashar IbrahimMagdalena WieczorekBassel E. SawayaMaha A ElbadryBeata Hukowska-SzematowiczMahdi Muhammad MoosaBeata KomorowskaMaher Al RwahnihBeate M. KümmererMahmoud KandeelBeatrice MacchiMahmoud Mohamed NaguibBeatrice MercorelliMahmoud NaguibBeatriz Escudero-PérezMahmut SafakBeatriz Vidana MateoMai IzumidaBee Kang TanMaite MuniesaBegoña SotMaite VaslinBen BerkhoutMaja M. LunarBen HauseMaja PotokarBenedikt KauferMajid KazemianBenjamin BaillyMakoto OzawaBenjamin ChenMakoto TakedaBenjamin DewalsMalachy Ifeanyi OkekeBenjamin LampMalcolm McConvilleBenjamin MeyerMalgorzata ŁobockaBenjamin OrsburnMałgorzata Polz-DacewiczBenjamin TrinitéMałgorzata Pomorska-MólBenoit VisseauxMana ItoBernard ChinaMana RaoBernard KaićMandy MullerBernard KlonjkowskiManel Essaidi-LaziosiBernard La ScolaManfred HeinleinBernard MossManfred MarschallBernhard ReschManfred WeidmannBert RimaManish SagarBert SchepensManon NayracBertram FlehmigMan-Seong ParkBertrand PainManuel Claude PeitschBeth A. GarvyManuel Gomez Del MoralBethany BollingManuel José Rodríguez OrtegaBhagirath SinghManuel Martínez-GarcíaBhaja Krushna PadhiManuel Méndez-BailónBharat B. AggarwalManuela SironiBianca SchulteMarc DesforgesBianchi LucaMarc FuchsBijal ParikhMarc GirardBin LiMarc GüellBingchun ZhaoMarc JaminBingqian QuMarc JohnsonBinhua LiangMarc KvansakulBjörn Erik Ole JensenMarc LütgehetmannBlair StrangMarc SitbonBluma G. BrennerMarçal Pastor-AngladaBo LiangMarcel MuellerBo Ram BeckMarcela LizanoBo WangMarcela RodrigueroBodo PlachterMarcello CandelliBogumil BryckiMarcelo SoaresBon-Sang KooMarcin OsuchowskiBorek SehnalMarco CiottiBorodynk-Filas NataszaMarco IannettaBoucau JulieMarco MerliBożena Nejman-FaleńczykMarco Yung-Cheng LinBrad GilbertsonMarco ZuinBradford K. BergesMarcus KaulBradley GrovemanMarek AsmanBrajesh SinghMarek KochańczykBrandon KeeleMarek KowalczykBratko FilipičMarek PetrášBreno De Mello SilvaMarek SmiejaBrent LehmanMarek WideraBrent RyckmanMaren EggersBrett L. HurstMargaret Adele ScullBrian BothnerMargaret J. LangeBrian C. SchaeferMargaret KielianBrian JordanMargaret M HannanBrian WillettMargarida DuarteBrigitta LaksonoMargarita Pons-SalortBrigitte Biesinger-ZwostaMargherita DessoleBruce A. DavidsonMargo BrintonBruce SmithMaría J. García-MurriaBruce TorbertMaría A. AyllónBruno PradinesMaria Amparo AssisBruno VaslinMaria Angelica GuimarãesBryan S. KaplanMaria Cássia Mendes-CorreaBryce M. WarnerMaría CorreaBryce WarnerMaria Cristina De RosaBurke JamesMaría Del Rosario Morales EspinosaByron CaugheyMaria Elena MarcocciC. Dustin RubinsteinMaría Eugenia GonzálezC. Joaquin CaceresMaria FioreC. Thomas BockMaria Gonzalez-CaoCaijun SunMaria Grazia CusiCaitlin A. O’BrienMaria I. GiraldoCaitlin MullarkeyMaria J. MaciasCălin CăinapMaria JenckelCalogero DiBellaMaria José AmengualCamelia ProtopopescuMaria K DahleCamilla TincatiMaria LucaCamille SureauMaria MasucciCamus ChristelleMaria PajunenCarina CarvalhoMaria PerdomoCarina ReuscherMaría Rosa López-HuertasCarissa Embury-HyattMaria RosenthalCarl DeomMaria Salomé GomesCarl KirkwoodMaria SalvatoCarla Bianca Luena VictorioMaria Serena BeatoCarla FontanaMaria Söderlund-VenermoCarla MirandaMaria TempestaCarlo AlfieriMaria Teresa SciortinoCarlo BuonerbaMaria TotanCarlo CervellatiMaria TrovatoCarlo RodolfoMariana MarinCarlo SantambrogioMariana PilottoCarlo V. CitterioMariangela FiginiCarlos AriasMarie Angélique De-ScheerderCarlos De NoronhaMarie GallouxCarlos EscalanteMarie HagbomCarlos G. Das NevesMarie UmberCarlos P. RubbiMarie-Anne Rameix-WeltiCarmela ConteMarie-Edith LafonCarmelo RapisardaMarie-Theres PohlCarmen CerchiaMariette DucatezCarmen Elena GomezMarij J.P. WeltersCarmen López-IglesiasMarija BackovicCarmen RivasMarijana StojanovicCarmina GallardoMariko MizuguchiCarmine FinelliMarilisa NovaccoCarol Chitko-MckownMarilyne UzestCarole HéniqueMarina BobkovaCarolina AriasMarina LusicCarolina RosadasMarina StrakhovskayaCarolina SimioniMarina StukeljCarolina StenfeldtMarine WasniewskiCarolina Susana CerrudoMario CannataroCaroline C. CordeiroMario HabekCaroline DemeretMario MietzschCaroline ScholtesMario PerkovicCaroline TapparelMario PlebaniCarolyn A. LarabellMariola DutkiewiczCarolyn CoyneMarion DesdouitsCasey Barton BehraveshMarion EnglandCasper RokxMarisa GariglioCassandra GuarinoMarisa GranatoCassandra KohMaritza N. Puray-ChavezCatarina MilhoMarius MasiulisCaterina Maria GambinoMariusz StasiolekCaterina VicidominiMark G. HindsCatherine EichwaldMark J. FersonCatherine M BrownMark PandoriCathia SoulièMark StengleinCécile BreytonMark ZaninCécile PhilippeMarkus GlätzelCecilia CalabreseMarkus KellerCedric LeyratMarla BerryCeleste DonatoMarlene DreuxCeline BressolletteMarlies BallegeerCéline Marie Elise GossnerMarta CanutiCelso CunhaMarta De AngelisCeri FieldingMarta GiovanettiChad RoyMarta L. DeDiegoChad SwansonMarta LuigiChan Kuan RongMarta Maria GagliaChang-Chi LinMarta MassanellaChangjun GuoMarta MonzónChanhee ChaeMarta S ShocketChantal VogelsMarta VallinoChantelle AhlenstielMartha BrownChao WuMartha I. NelsonChao-Nan LinMartin A. ErlandsonChaoping ChenMartin BartasCharalampos SiristatidisMartin ChanCharith Raj Adkar-PurushothamaMartin D. RyanCharles BanghamMartin DaveyCharles E. SamuelMartin DruckerCharles El HageMartin HengesbachCharles GroseMartin HeßlingCharles H. CalisherMartin HölzerCharles ReynardMartin MesserleCharles RupprechtMartin PelchatCharles StauftMartin R. GoodierChaturaka RodrigoMartin RafteryChe C. ColpittsMartin SappChee Keng MOKMartin ScholzCheng HuangMartin TolstrupCheng-Fang YenMartina KochChenglei LiMartina SalákováChen-Hua LiuMartine AubertChetan MeshramMartine BraibantChiaho ShihMarvin EdeasChian-Shiu ChienMarwa HanafiChiao-Fang TengMarxa L FigueiredoChiara ChiozziniMary BeilbyChiara DentoneMary CasertaChiara Di TucciMary F. KearneyChiara FornaraMary HittChiara MedagliaMary Katherine MillsChiara PinoniMary Lea KillianChiara PiubelliMary N. SheppardChiara PontremoliMarylène MougelChiara TurchiMary-Louise PenrithChichun HoMarzena Rola-ŁuszczakChi-Chun HoMasaaki MiyazawaChi-Chung ChouMasahiro NiikuraChienjin HuangMasaki ImaiChihong SongMasanori AtsukawaChih-Wen PengMasaru ShimadaChihyang WangMasashi NinomiyaChing Lung LaiMasayuki SaijoChinmay Kumar MantriMasmudur M. RahmanChin-Mu HsuMassimo BiagettiChin-Tien WangMassimo CiccozziChi-Wei TsaiMassimo FiorilliChloé DiMeglioMassimo PieriChris RobinsonMassimo PizzatoChris VerhofstedeMathias LichterfeldChristian CambillauMathias MartinsChristian CordanoMathias SchemmererChristian De La FeMathieu DubéChristian GrundMathieu J.F. CrupiChristian H EggersMathieu MateoChristian NapoliMathilde RichardChristian PetersMatteo BolcatoChristian PfallerMatteo LegnardiChristian SiatkaMatteo NegroniChristian WillyMatteo RiccòChristiane RiedelMatthew CollinsChristiane Weissenbacher-LangMatthew D. MooreChristina B. KarstenMatthew KnoxChristina GuzzoMatthew L. BochmanChristina Hernández-MunainMatthew MilhollandChristina HutsonMatthew R. ReynoldsChristina KitchenMatthew ReevesChristina LeysonMatthew TaylorChristina M. NewmanMatthias NiedrigChristina StraubMatthias ReddehaseChristine A. KingMatthias SchweizerChristine BurkardMattia RiefoloChristine E. EngelandMaureen H.V. FernandesChristine Fehlner-GardinerMaureen TaylorChristine HappelMauricio Comas-GarciaChristine M. CaltonMaurizia Rossana BrunettoChristine PourcelMaurizio FedericoChristine SzymanskiMauro PistelloChristoph BuchtaMax L. NibertChristoph BurckhardtMax RennerChristoph KreerMax Von KleistChristoph Neumann-HaefelinMaxim D. SeferovicChristoph SpinnerMaya DymovaChristophe GuillonMaya WardehChristopher AikenMayke LeggewieChristopher ColemanMayumi KomineChristopher HellenMayur ParmarChristopher L. NethertonMazahar MoinChristopher LupferMeehyein KimChristopher StobartMegan LloydChrysoula OrfanidouMegan O'ConnorChrysovalantis KorfitisMegan SpurgeonChuan ChenMegha AggarwalChuen-Fu LinMehdi EmamChukwunonso OnyilaghaMehdi KabaniChun-An ChengMei-Ling LiChunchun MengMelania PudduChungchi HuMelina FischerChunhe GuoMelinda BrindleyChunhong DongMelissa Bello-PerezCica UrbinoMenira SouzaCinzia CaudaiMeret E RicklinCiro IsidoroMeritxell García-QuintanillaCiro SalzanoMette MyrmelClaire DebackMichael A. PurdyClaire MuslinMichael AlkanClaire Shannon LoweMichael BukrinskyClara Torres BarceloMichael BuschmannClarissa M. KochMichael HolbrookClaude KrummenacherMichael HuberClaude RispeMichael J. MelzerClaude SabetaMichael JukesClaudia ColombaMichael KhachayClaudia Fernández-AlarcónMichael KoldehoffClaudia FortunaMichael LetkoClaudia Maria TucciaroneMichael M.C. LaiClaudia MatteucciMichael McVoyClaudia WylezichMichael MullanClaudio De LiberatoMichael NassalClaudio FeniziaMichael RaheClaudio GambardellaMichael RajnikClayton WinklerMichael RiehleColette PeitzschMichael RocheColin ButtimerMichael RoggendorfColleen JonssonMichael S. SeamanCon SullivanMichael SchindlerConcetta CastillettiMichael SchreiberConnor BamfordMichael ThemisConrad FreulingMichael WilkinsonConrad Martin FreulingMichaela RumlováConstantinos S. KyriakisMichal HetmanCoretta Van Leer-ButerMichal KuklaCorey L. CampbellMichal LinialCorinne Schmitt-KeichingerMichal PyzikCornelia HeinzeMichal ToborekCornelia SpethMichel PépinCornelis J.M. MeliefMichela DeianaCorrado AngeliniMichele CameroCorrado PelaiaMichele GiannattasioCory KnudsonMichele LunardiCostin-Ioan PopescuMichèle OttmannCraig BlackstoneMichele TonelliCraig BrunettiMichelle GroomeCraig CameronMichihito SasakiCraig WebsterMichiko MatsushitaCriscuolo ElenaMidori TabaraCristina CelmaMiguel A. MartínezCristina DomingoMiguel Ángel Jiménez-ClaveroCristina Mas-BarguesMiguel Angel Muñoz-AlíaCristoforo PomaraMiguel Ángel Rodríguez-MillaCrystal A. MendozaMiguel CoronaCurtis BrandtMiguel FernándezCynthia A. Pise-MasisonMiguel Julián Martínez YoldiCyprian C. RossettoMiguel MartinsCyril Le NouenMiha SkvarčCyrille MathieuMihayl VerbanovD. MuthuchelvanMihnea BostinaDachrit NilubolMike TsionasDae-Hyuk KimMike Yw KwanDagmara BialyMikeljon NikolichDainius ZieniusMiki ImanishiDaisy HoaglandMikio SuzukiDaisy StaintonMilan SencanskiDaisy W. LeungMiles TheurerDamià GarrigaMilka MilevaDamien TullyMinetaro AritaDamu TangMing Kun HsiehDana H. GabuzdaMing Te YehDana L. VanlandinghamMing XiaDana VanlandinghamMing-Ching HsiehDaniel BonthiusMing-Chu ChengDaniel CohenMinghang WangDaniel De VosMing-Jing HwangDaniel Enosi TuipulotuMing-Kun HsiehDaniel FadenMinglei GuoDaniel GeraghtyMing-Wei LuDaniel GoldhillMinhua LuoDaniel J. RawleMinkyung YiDaniel L. RockMinna PoranenDaniel LingwoodMira A.M. BehnamDaniel MachinMiranda De GraafDaniel MacqueenMireille AnsaldiDaniel MarcMiri KrupkinDaniel MeltersMiria CriadoDaniel N. BolonMiriam KlausbergerDaniel NicholsMirjam B. ZeiselDaniel P. PotaczekMirko TrillingDaniel Pérez-NúñezMiroslav GlasaDaniel Sanchez-TaltavullMiroslaw GilskiDaniel StoneMirosław Paweł PolakDaniel TodtMisa KorvaDaniel WolffMitsuoki KawanoDaniel WrightMitsutoshi YoneyamaDaniela CalinaMizuki YamamotoDaniela De VitaModesto Redrejo RodriguezDaniela LoconsoleMohamed Abdo RizkDaniela MalatestaMohamed DahaDaniele ContiniMohamed Ismail AshrafaliDaniele FocosiMohamed M. El-GazzarDaniele LapaMohamed-Elamir F. HegazyDanielle E. AndersonMohammad Al-HatamlehDanilo Oliveira CarvalhoMohammad HajizadehDann TurnerMohammad Hassan BaigDanushka WijesundaraMohammed A. RohaimDarius KazlauskasMohammed RohaimDarpan ChakrabortyMohan AmarasiriDarryl FalzaranoMohan Kumar Muthu KaruppanDasja PajkrtMohan MaheshDave SpeijerMohd ShahbaazDavid A. BenfieldMohsan SaeedDavid BhellaMohsin JafriDavid BoutolleauMoises DiagoDavid DuniganMoises Leon-JuarezDavid E. StallknechtMojtaba EhsanifarDavid EcclesMonia MarchettiDavid EvansMonica BassoDavid G. HeckelMonica G. CandelaDavid GarfinkelMonica GiammarioliDavid HawmanMonica Malone McNealDavid HughesMonika Adamczyk-PopławskaDavid J. SpiroMonika LindemannDavid Jesse SanchezMonika Pazgan-SimonDavid KingsleyMonika RinderDavid KnudsenMonika ZelazowskaDavid LesbarresMonique BeuveDavid MarchantMonique LafonDavid Margulies MarguliesMonique VogelDavid McVeyMookkan PrabakaranDavid NegusMorgan BrisseDavid OrnellesMorgan CraigDavid SchultzMoriah SzparaDavid SmithMoritoshi IwagamiDavid SteffenMorten PedersenDavid VerhoevenMosè ManniDavid VermijlenMotomu ShimaokaDavide AngelettiMotomura KazushiDavide PacificoMounira K. Chelbi-AlixDean D. ErdmanMozhdeh ZamaniDean KonjevicMrigendra K.S. RajputDebbie McKenzieMrinmoy GhoshDebora LanznasterMudit TyagiDeborah KarasekMuhammad IlyasDeepak B. Thimiri Govinda RajMuhammad Kashif SaleemiDeepak ShuklaMuhammad MunirDelphine MuriauxMurali MunirajuDemetra S.M. ChatzileontiadouMurugan SubbiahDemócrito De Barros Miranda-FilhoMutien-Marie GariglianyDenis GerlierMutsuyo Takayama-ItoDenis KainovMya Myat Ngwe TunDenis KolbasovMyriam ErmonvalDenis LeclercMyrna Cristina BonaldoDenise MarstonMyrna M. MillerDenise MeyerMysore R. SudarshanaDenys A. KhaperskyyNadia MarascioDerek McMahonNadine LübkeDespoina BerisNaeem KhanDevadas KrishnakumarNaga Venkata Sabarinath NeerukondaDevendra Kumar RaiNagendrakumar Balasubramanian SinganalurDevika SirohiNagesh KolishettiDeyu XieNai-Huei WuDhanjai DhanjaiNan HaoDharmendra K. YadavNancy C. HortonDi LiuNancy H.L. LeungDiana J.M. Van Den WollenbergNancy Weiland-BräuerDiana MartinsNaomi ForresterDiana ScorpioNaoto FujiwaraDiana V. PastranaNaoto ItoDiane AddieNaphak ModhiranDiego AlvarezNatália FreitasDiego FerreroNatalia GolenderDiego ForniNatalia Mazur-PanasiukDieter BlaasNatalia Soriano-SarabiaDieter HoffmannNatalie E. NetzlerDimitrios SklirosNatalya GolenderDimitrios StakosNataša KnapDinesh Chandrasekaran DhurvasNatasha J. SharpDipti NayakNatasha N GaudreaultDirk De GraafNatasha N. GaudreaultDirk GrimmNatasha Tilston-LunelDirk MartensNathalie ChazalDirk WohlleberNathamon KosoltanapiwatDivya KalraNathaniel DenkersDmitriy V MazurovNathaniel M. ByersDmitry KostyushevNaveen VaidyaDmitry TworowskiNaveen VankadariDoina AtanasiuNaveenchandra SuryadevaraDomenico FrancoNazlı AyhanDomenico MaisanoNeeltje A. KootstraDomenico MattoscioNeil P. KingDomenico RizzoNeila SaidiDomenico SiricoNejat DüzgüneşDominic Paquin-ProulxNenad PandakDominic QualleyNenavath Gopal NaikDon GammonNetanel TzarumDon SodoraNeven PapicDonatella FerraroNiccoló VendraminDong Sung AnNicholas ChesarinoDonghoon ChungNicholas DopkinsDong-Hun LeeNicholas J. LennemannDongming ZhouNicholas JohnsonDonna A. MacDuffNicholas KomarDonna NeumannNicholas M. KiuliaDorian McIlroyNicholas M. ProvineDorota Zarębska-MichalukNicholas T. BelloDorothea BankwitzNichole BrinkmanDorothea K. ThompsonNichole HinesDorothee Von LaerNicla RomanoDouglas GladueNicola AbbresciaDouglas LeamanNicola CioffiDragan BrnićNicola DecaroDragos HorvathNicola F. FletcherDrucilla G. RobertsNicola FioreDuane P. GrandgenettNicolas BoisgeraultDylan LeiNicolas De ProstDylan M JohnsonNicolas GinetE. Dan HellerNicolas LévêqueE. SchvoererNicolas MeunierEdan TulmanNicolas RuggliEdeildo Ferreira Silva-JúniorNicole FischerEdiane SilvaNicole GrandiEdmond J RemarqueNicole N. HaeseEdmund KoziełNicole R. SextonEduardo F FrancaNicole VidalEduardo Gomez-CasadoNicoleta AndreescuEdward DuboviNicoletta CasartelliEdward P. BrowneNicoletta UrbanoEdward StephensNicolò MussoEdward TrybalaNídia Sequeira TrovãoEdyta ŚwiętońNiels A W LemmermannEiichi SasakiNiels LorenzenEike SteinmannNiels PiotEiko MatsuoNikolai S. ProkhorovEili HuhtamoNikolaos GrigoriadisEkaterina A. KorotkovaNikolaos PerakakisEkramy SayedahmedNikolas FriedrichElba MaurizNikon VassilakosElena BencúrováNilakshi BaruaElena CicheroNils Christian GassenElena CircellaNils GassenElena GregoNils LemmermannElena Marbán-CastroNina WolfrumElena ParianiNingguo FengElena PomariNisha DuggalElena Victorovna DeinekoNishit GoradiaEleni GolomazouNobumichi KobayashiEleni TzikaNobuo KanazawaEleonora PonterioNoelia Carmona-VicenteEli D. EhrenpreisNonhlanhla TlotlengEli EisenbergNor ChejanovskyEliana De LucaNora ChapmanEliette BonnefoyNorbert J. RobertsElif NurtopNorbert NowotnyElihu Aranday-CortesNorikazu IsodaElina LaantoNoriko OgasawaraElisa BolattiNuno S. OsórioElisa MartróNuno TaveiraElisa MazzottaNunzia LinzaloneElisa VisherNuria MontesElisa WirthgenNuria TornerElisabet PujadasOhad MazorElisabeth A. FalendyszOk Sarah ShinElisabeth Fichet-CalvetOlaf IskenElisabetta ManaresiOle Herman AmbúrElisabetta ProfumoOl'Ga A. TarasovaElisabetta RazzuoliOlga E. IvanovaElisenda BallestéOlga Lilia Garibay-CerdenaresElizabeth Ann L. EnningaOlga MatveevaElizabeth DraganovaOlga S. SokolovaElizabeth HenryOlga V. MorozovaElizabeth Ramirez-MedinaOliver FacklerElizabeth SpargerOlivier EscaffreElizabeth WhiteOlivier MauffretElizabeth ZumbrunOlivier NègreElke BognerOlivier ReynardElke HumerOlivier SchildgenElla SklanOlve PeersenEllen KrautkrämerOlympia E. AnastasiouElliot AndrophyOmbretta TurrizianiElmostafa BAHRAOUIOndrej CinekElodie Monchatre-LeroyOndřej LenzEloi R VerrierOran ErsterEl-Sayed Abdel-WhabOren KobilerEl-Sayed M AbdelwhabOrkide O. KoyuncuElsebeth LyngeOrsolya KutasiElton J. R. VasconcelosOscar Hurtado GonzalesElzbieta SarnowskaOscar Hurtado-GonzalesEman AnisOtávio EspíndolaEmanuel GoldmanOttmar HerchenröderEmilia HadziyannisOtto HallerEmilien JeannotOuti LyytinenEmily A HemannOwen HigginsEmily B. HollisterOya CingozEmily Desmet LedgerwoodPablo CaballeroEmily S.J. EdwardsPablo GastaminzaEmma GrantPablo GonzálezEmma L. MohrPablo Guardado-CalvoEmma L. WisePäivi Ylä-AnttilaEmmanouil N. MagiorkinisPakieli H. KaufusiÉmmanuel BréardPakorn AiewsakunEmmanuel GordienPalaniappan RamanathanEmmanuel J. FavaloroPamela Di GiovanniEmmanuel RoilidesPamela Martinez-OrellanaEndler LukasPanagiotis G. HalvatsiotisEnikő FehérPanagiotis PeitsidisEnnio ProsperiPanayampalli Subbian SatheshkumarEnric MateuPaola Dall’AraEnrica ZuccaPaola De BenedictisEnrico CarusoPaola Del PortoEnrico GrandePaola J. PérezEnrico LavezzoPaola VenierEnrico PavoniPaolo CavorettoEnrique MorionesPaolo ConflittiEran BacharachPaolo Emidio CrisiEric AgboliPaolo GiuffridaEric BortzPaolo MottaEric C. FreundtPaolo PastorinoEric ChampagnePaolo ZaffinoEric De Souza GilParmit SinghEric DelaportePascal MiesenEric DuboisPascal MutzEric DykemanPasquale SaldarelliEric FreedPatricia A NuttallEric GowansPatricia A ThibaultEric HanssenPatricia BerthonEric J. SchottPatricia DevauxEric MosselPatricia LiWangÉric NiqueuxPatricia PesaventoEric PoeschlaPatricia PriceEric S. HoPatrícia SerraEric SchirmerPatrick ArbuthnotEric YagerPatrick BronErik Jurjen Duintjer TebbensPatrick DolanErin E. SchirtzingerPatrick LabonteErin Elizabeth SchirtzingerPatrick McClureErkko YlösmäkiPatrik EllströmErnest A. GouldPatrizia FarciErnest BaileyPaul A BrownErnesto QuesadaPaul A. RotaErsheng KuangPaul GustafsonErxun ZhouPaul HickEsmeralda MinguijonPaul J LehnerEspen RimstadPaul J. NormanEsperanza Gomez-LuciaPaul J. Wichgers SchreurEstanislao Nistal-VillánPaul KinchingtonEsteban Hernandez-VargasPaul L. GuyEstela Escribano-RomeroPaul LehmannEstelle VenterPaul McCrayEster Leno-DuránPaul MurrayEsther BullittPaul OesterleEsther Dawen YuPaul RossiterEsther MahabirPaul SpearmanEthan L. MorganPaul ValleEtsuro ItoPaul YipEun Jeong ParkPaulo Ricardo Martins-FilhoEva Böttcher-FriebertshäuserPaulraj KanmaniEva KriegovaPavel GershkovichÉva NagyPawel BieganowskiEva-Maria MittlerPawel ZmoraEvaristo Vázquez-DomínguezPawin PadungtodEve PécheurPedro GarcíaEvelyne P. Picard-MeyerPedro Gómez LópezÉvelyne SchvoererPedro J. AlcoleaEverardo VegaPeifang SunEvgeny KulikovPei-Jer ChenEvžen BouřaPeilin ZhangEwa BrzozowskaPei-Shi YenEwa DudzińskaPei-Yun ShuEwa Jończyk-MatysiakPellini RaulEwa PlantPeng ZhouEwa TalarekPengxiang ChangEwa TomaszewskaPengxing CaoFabian B. FahlbuschPenmetcha KumarFabian Z.X. LeanPenny ClarkeFabiana SupertiPenny P. PowellFabien P. BlanchetPerminder GulaniFabio RomerioPernille HolmagerFabio ScarpaPetar KnezevicFabiola Elizabeth VillanovaPeter A. DurrFabiola MartinPeter GaskillFabrizio BrontePeter HalfmannFabrizio CilloPeter HemmerichFabrizio MaggiPeter KirklandFabrizio PucciPeter KubiszFaizal Abdul CareemPeter MastrangeloFaizal CareemPeter MedveczkyFangfeng YuanPeter NevilleFang-Tzy WuPeter PalukaitisFanny LegrandPeter PelkaFarooq NasarPeter RevillFaten A. OkdaPeter Van Der VoortFatma BerriPeter VrsanskyFayna Diaz-San SegundoPetr HorinFazlurrahman KhanPetr KomínekFederica MagagnoliPetra StrakováFederico BertoglioPetras PrakasFederico GiannittiPetrus Jansen Van VurenFederico PapaPhaneendra K. DuddempudiFedora GrandePhilip El-DuahFeilong WangPhilip John LesterFelice LorussoPhilip TedburyFelipe Diaz-GrifferoPhilip V’kovskiFelix BroeckerPhilipp KolbFelix NjeumiPhilippe AfonsoFeng LiPhilippe ColinFengmin LuPhilippe CotelleFerdinand RoeschPhilippe RascleFerman ChavezPhillip C. GaugerFernando BauermannPhu DuongFernando CardonaPia GattingerFernando FerreiraPierre BessièreFernando Gonzalez-CandelasPierre RoquesFernando OsorioPierre-Emmanuel JoubertFernando RodriguezPierre-Simon RohrlichFernando Torres-PerezPierre-Yves LozachFielding Burtram ClintonPieter LibinFinn GreyPieter-Jan D. GunsFlor H PujolPietro FerraraFlora De ContoPil Soo SungFlorence BuseynePin LingFlorence LarrousPin-Nan ChengFlorian PfaffPio ContiFlorin George HorhatPiotr GolecFlorine ScholtePiotr TrzonkowskiForgia MarcoPo-Huang LiangFranca FagioliPoli GuidoFrancesca Di GiallonardoPooja SaxenaFrancesco Andrea ProcopioPool EdmundFrancesco CoreaPo-Yuan KeFrancesco Di GennaroPradeep D. UchilFrancesco FelizianiPragya YadavFrancesco MessinaPramod K. SahuFrancesco MiraPrana V. ShahFrancesco NazziPranshu SahgalFrancesco NegroPrasad ParadkarFrancesco NicoliPrashant DesaiFrancesco SimonettiPrashant SharmaFrancesco SpinozziPrasun K. DattaFrancis DelpeyrouxPraveen P.N. RaoFrancisca SamsingPrema Latha MallipeddiFrancisco Campello Amaral MelloPrenilla NaiduFrancisco EpeldePritam PandaFrancisco LlorentePriya LuthraFrancisco Rodriguez-ValeraPyrear Suhui ZhaoFranco MutinelliQiang LiuFrancois MeurensQiangmin ZhangFrançois MeurensQiangming SunFrançois-Loïc CossetQibin LengFrançois-Xavier BriandQihan LiFrane PaicQin FangFrank JenkinsQinfeng HuangFrank OechslinQingbo LiuFrank WongQiuhong MiaoFranklin NobregaQiwei QinFranklin NorbregaQiyi TangFranklin Wang-ngai ChowQuaiser SaquibFranklin W.N. ChowR. Eduardo PalmaFranz-Xaver SewaldRachael E. TarlintonFraser HillRachael TarlintonFrauke MueckschRachel PalinskiFred EyaseRachel Van DuyneFred L. HomaRachid MazrouiFrédéric FarnirRachy AbrahamFrederic Le GalRadha GopalFrederic SchoenbergRadu BotgrosFriederike ReussRadu Claudiu FierascuFriedhelm PfeifferRafael DelgadoFuh-Jyh JanRafael J. VillanuevaFulvio MarsilioRafael Jacinto VillanuevaFumihiro KatoRafael Villalba-MontoroFun-In WangRafaela Salgado FonteneleG. SancesarioRaffael NachbagauerG. W. Gant LuxtonRaffaele De FrancescoGábor FöldváriRaffaele La PortaGabriel Del RioRaffaele StrippoliGabriel GonzalezRagunath SingaraveluGabriel J. StarrettRagunathan DevendranGabriel ParraRahul NelliGabriela Tomas JerônimoRaimunda Do Socorro Da Silva AzevedoGabriele FuchsRajendran RajeswaranGabriele LandoltRalf BartenschlagerGabriele PaganiRalf WagnerGabriella D’EttorreRalph FeuerGabrielle VieyresRalph G. DepalmaGaël ThébaudRamces Falfan-ValenciaGaelle GonzalezRamesh GoelGaëtan GuignardRamiro IsazaGaetan LigatRamon AlemanyGaetano GalloRandal N. JohnstonGaetano ThieneRandall J. CohrsGajanan N. SapkalRandall RenshawGannon C.K. MakRangmar GoelzGareth BradyRanieri BizzarriGarilyn M. JentarraRaquel Bello-MoralesGary AnRaquel HernandezGary C. AnRaquel LemeGary MartyRaquel TenorioGary McLeanRashid ManzoorGary WongRavi DissanayakeGaurav Raj DwivediRavindra KumarGautam SethiRay YokomiGema Mendez-LagaresRaymond BujdosoGeoff HolmRazia ShaikGeoffrey HutinetRebecca BrocatoGeorge B. KyeiRebecca DuBoisGeorge BelovRebecca SkalskyGeorge CarnellRebekah C. KadingGeorge G. KennedyRebekah R. HeltonGeorge P. LomonossoffReed F. JohnsonGeorge ValasoulisRegina G. KleespiesGeorges HerbeinReiner UlrichGeorgia BraliouReinhard ErtlGerald Fritz SchusserReinhild PrangeGerald HeckelReinout Alexander BemGéraldine PiorkowskiRemi N. CharrelGéraldine Schlecht-LoufRemigiusz WorchGeraldine TaylorRenata GrzywaGergana G. ZahmanovaRenata ŽunecGerhard DoblerRené C. OlsthoornGerlind SulzenbacherRenee KingGerlinde Van De WalleRenée M. Van Der SluisGerman PerezRenfeng LiGerry McLachlanRenuka RamanGert ZimmerRenzo BoldoriniGerwin HellerReza KhayatGhazi KayaliRicardo B. LeiteGheorghe IliaRicardo ParreiraGiada MattiuzzoRicardo Soto-RifoGian Mario CossedduRichard BinghamGian Paolo CavigliaRichard BowenGianfranco LaulettaRichard DixGianluca Di MiccoRichard Edward RandallGianluca VergineRichard H. ScheuermannGianmaria D'AddazioRichard J. RollerGianpaolo CoroRichard J.P. BrownGianvito LanaveRichard LeeGijs VersteegRichard MalikGilbert VerbekenRichard PaulGilberto Sabino-SantosRichard SmithGilles GadeaRichard StantonGIna SuhRichard WhitleyGiorgio GambinoRichard YanagiharaGiorgio ManginoRichard ZimmermannGiovanni CiliaRik De SwartGiovanni CristalliRikio KirisawaGiovanni FilocamoRikiya TakeuchiGiovanni FranzoRina HamajimaGiovanni MariniRob A. GrutersGisela MasachessiRob HoebenGisselle N. MedinaRob WhiteGitta TurowskiRobert A. KozakGiulia FranzoniRobert C. CarlisleGiulia Freer Robert CzajkowskiGiuliano FurliniRobert DrillienGiuliano RamadoriRobert FlisiakGiulietta VenturiRobert GiffordGiuseppe BertoniRobert HarrisonGiuseppe BertozziRobert HarrodGiuseppe CardilloRobert HertelGiuseppe LegnameRobert J. FischerGiuseppe MurdacaRobert L. GottliebGiuseppe RivaRobert McLeanGiuseppe TarantinoRobert PosseeGiusy CardetiRobert SilvermanGlaucia Paranhos-BaccalàRobert SmithGodwin W. NchindaRobert V. StahelinGoedele MaertensRobert Van DomselaarGoncalo SilvaRobert WatsonGonçalo SilvaRobert Yung-Liang WangGoo-Bo JeongRoberta BruhnGopas JacobRoberta LecisGöran LarsonRoberta MancusoGordana Wozniak-KnoppRoberta PeregoGourav BhardwajRoberta RizzoGraça PintoRoberto LilliniGraham BelshamRoberto P. GarofaloGraham BrogdenRoberto PiacentiniGraham FreimanisRoberto Vicente Gozalbo RoviraGraham HatfullRobin KettelerGraham TaylorRobin StanleyGraziana Da RoldRobin WarneGrazyna LietzauRocco PiazzaGrażyna Majkowska-SkrobekRod RussellGreg BrennanRodolphe BarrangouGrégory DubourgRodolphe HamelGregory J. FonsecaRoenick Proveti OlmoGregory J. RobertsonRoger FrutosGregory LambertRohan SharmaGregory MelikianRohini DattaGriffith ParksRohit JangraGrigoris AmoutziasRoland SchwarzerGrigoris ZoidisRoland ZellGrzegorz PanasiewiczRolesu SandroGrzegorz TeresińskiRollie J. ClemGrzegorz WegrzynRomain RouetGrzegorz WoźniakowskiRomain VolleGualtiero AlvisiRomain Vuillefroy De SillyGuangai XueRoman KandarGuangdi LiRoman NowickiGuanghui WuRoman PrymulaGuangming RenRomina SalpiniGuang-Wu ChenRonald B. TurnerGuangyu LiRonald HartyGuangzhao LiRonald S. VeazeyGuerrino MacoriRonald SwanstromGuidenn SulbaranRong HaiGuido VanhamRory HendersonGuillaume A. DurandRosa Coldbeck-ShackleyGuillaume BeauclairRosa GálvezGuillaume CarissimoRosa M PintoGuillaume CastelRosa María Del AngelGuillaume CrovilleRosa MurilloGuillaume GiraudRosaria ArviaGuillaume MittaRosemarie M. BoozeGunnel HalldenRossella SartoriusGunnveig GrodelandRoy A. HallGünter KampfRoya ZandiGuntur FibriansahRuben Hernandez-AlcocebaGuochun JiangRuchika BajajGuodong RaoRui LuoGuozhen LiuRuifang WeiGuozheng WangRuifeng HuGurdeep SinghRuimin GaoGustavo DelhonRuiz AutumnGuy LemayRuklanthi (Rukie) De AlwisGyőző L. KajánRushika PereraHabiba AlsafarRussell J. DiefenbachHachung YoonRūta KananavičiūtėHae-eun KangRuth D. FritschHaidar AklRuth Serra-MorenoHaidong GuRuth TachezyHana DrahovskáRuyin LiuHana M. DobrovolnyRyan TroyerHana ZelenáRyo OkadaHanki LeeRyoyo IkebuchiHanmant GaikwadRyszard KierzekHans HenkesRyuichi SugiyamaHans-Georg RammenseeRyuta UrakiHansjörg LehnherrS. Layton DanielHany AnanySabine BrandtHapuarachchige Chanditha HapuarachchiSabine Chapuy-RegaudHarald WodrichSachiko KoyamaHarini SooryanarainSadjia BekalHarriet GroomSaeid GhavamiHarshad IngleSaeid Najafi FardHarvey M. FriedmanSafder GanaieHasan EjazSaija KiljunenHashimoto KoichiSaikat BalaHassan MahsoubSaikat BoliarHatem A. ElshabrawySajjad AhmadHéctor HidalgoSajjad ShiraziHector MorenoSakshi AroraHee-Chun ChungSally MolloyHeejoon MyungSally R. RobinsonHeidi ContrerasSally RobertsHeiko HerwaldSalman HussainHeinz ZeichhardtSamantha J.M. EvansHejun LiuSamantha KendrickHelen CrookeSamina AlamHelen DolkSamir GorasiyaHelen FarrellSamita AndreanskyHélène DutartreSampa MaitiHélène SanfaçonSamuel ConstantHeli HarvalaSamuel K. CamposHelle Bielefeldt-OhmannSamuel Ken-En GanHelmut FickenscherSamuel KilcherHenk-Jan SchuurmanSamuel Pushparaj Robert JeyasinghHenri GruffatSanchari BhattacharyyaHenriette De ValkSandeep DhandaHenrik PaavilainenSandeep Kumar VashistHenry FechnerSandeep UpadhyayHeribert StoiberSandino Ana MaríaHerman EgberinkSandra Blaise-BoisseauHerman FavoreelSandra DiederichHervé PouletSandra FrabasileHeui-Soo KimSandra JunglenHeverton DutraSandra PintoHidefumi KohSandra Rodriguez-PeralesHideki AizakiSandrine A. LacourHideki HayashiSandrine Valsesia-WittmannHideo KatoSandro KlafackHideto FukushiSandy EldridgeHie-Won HannSang Guen KimHimanshu GargSang Heui SeoHin Hark GanSangita SinhaHinh LySanjay MaggirwarHiroaki OkamotoSanjay RathodHiroaki WakimotoSanjay ReddyHirokazu KimuraSankar BasuHiromitsu MoriyamaSanna Maria SillankorvaHiroshi KatohSanne TerrynHiroshi ShimodaSanthi DevasundaramHiroteru KamimuraSantosh GeorgeHiroto ShinomiyaSantosh KumarHiroyuki ShimizuSara BaldassanoHisashi AkiyamaSara BoboneHitomi KinoshitaSara CapponiHo Yin Eric LauSara De MartinHollis O'NealSara FedericiHolly R. HughesSara MoutaillerHolly RamageSarah CunzeHomayon GhiasiSarah FrançoisHongping WeiSarah K. WoottonHongxuan HeSarah L. KempsterHoracio Andres Vargas GuzmanSarah LaMereHorst VogelSarah LondriganHossain FaridSarah M. DooreHovakim ZakaryanSarah R HaileHsin-Fu LiuSarah Van TolHuaji QiuSaravanan Subramaniam SubramaniamHugh DevlinSarka NemeckovaHuibin LvSarmila TandukarHuigang ShenSascha Y. KupkeHui-ling ChiouSasha R. AzarHui-Min ChungSatinder DahiyaHuitao LiuSatoko KanematsuHumberto F. BoncristianiSatoru AraiHumberto Lanz-MendozaSatoru WatanabeHung-Jen LiuSatoshi InoueHung-Sheng ShangSatoshi SekiguchiHung-Yao HoSatoshi TaniguchiHung-Yi WuSatu HakanenHURNG-YI WangSatu HepojokiHusni ElbaheshSatu MäkeläHussein AlySaurabh GautamHye Jin ShinSaveez SaffarianHye Kwon KimSaverio PaltrinieriHyeMee KimSchuyler Van EngelenburgHye-ra LeeScott G. MitchellHye-Ryoung KimScott LovellHyoung-shik ShinScott ReidHyuk-Joon KwonSD RabkinHyun Jeong KimSe Chang ParkHyun Jin KwunSeason S. WongHyunbo ShimSébactien HantzHyun-Jeong KoSebastian GisderHyun-Jin ShinSebastien GibotHyun-Woo ParkSébastien LemireIan BrierleySébastien LhommeIan HampsonSebla B. KutluayIan PattersonSeiichi KitagawaIan TietjenSeik-Soon KhorIbolya AndrásSeil KimIbrahim BozgeyikSeishi MurakamiIbrahim M. SayedSeki MasafumiIgnazio CaruanaSelmir AvdicIgor JurakSenol PiskinIgor RouzineSenthil ChinnakannanIgor TvaroskaSeongbin ParkIgor V. BabkinSerafin GutierrezIkbal Agah InceSerena BianchiIkuo ShojiSerena RobettoIlaria Di BartoloSergei NekhaiIlda D'AnnessaSergei Nikolaevich ShchelkunovIlija BrizićSergei SukharevIlka EngelmannSergey IordanskiyIlker IskenderSergey MorzunovIna BalkeSergey NetesovIna P O'CarrollSergi Padilla-ParraIndre Kučinskaite-KodzeSergio E. RodriguezInhee ChoiSergio Lo CaputoInmaculada FerriolSeth PincusInna ChabanSéverine HervéIn-Soo ChoiSeverino Jefferson Ribeiro SilvaIntawat NookaewShailesh KumarIntikhab AlamShan GohInTony AdesShannon KenneyIoannis A. GiantsisShanthi SabarimuruganIoannis GiantsisShaobo WangIoannis M. ZacharioudakisShaoli LinIolanda MangoneSharifah Syed HassanIoly Kotta-LoizouSharon BrookesIonela HoteaSharon ClouthierIpsita SahaSharon M BrookesIrene Lo CignoShauna KaplanIrene Ramos-lopezShawn BabiukIrfan RatherShawna McCallinIride Francesca CeresaSheikh Mohammad Fazle AkbarIrina Isakova-SivakSheila A. HaleyIrina KiselevaSheila GrahamIrina TikhonovaShelby O'connorIrit DavidsonSheli R. RadoshitzkyI-S. Wade HuangSheng-Fan WangIsabel Garcia-DorivalSheng-Wen HuangIsabel LegazShie-Liang HsiehIsabel Muñoz-BarrosoShigefumi OkamotoIsabella MarchesiShigeki SetoIsabelle CheminShigeki TakedaIsabelle DarbouxShih-Che SueIsabelle DietrichShih-Min WangIsaiah ArkinShihoko Komine-AizawaIsidoro MartínezShih-Yen LoIsmail SebinaShijin JiangIsrael PagánShin MurakamiIstván KissShin-Hong ShiaoItalo TemperaShinji OhnoItay RoussoShinji WatanabeIvan F.N. HungShintaro KobayashiIvan HirschShin-Yi MarzanoIvan KosikShiow-Her ChiouIvan TrusShiro FukutaIvana GrgićShitao LiIvana LazarevicShiv BharadwajIvana LojkićShoichi SakaguchiIvelin GeorgievShou-Wei DingIvone MartinsShouxiong HuangIwao KukimotoShrikant PawarIwona Markowska-DanielShu Chi WangIwona MorkunasShu ShenIzabela RaganShuai XiaIzabela ZakrockaShuetsu FukushiJ. Chad BrennerShuhei MiyashitaJ. David BeckhamShuming BaoJ. Peter GogartenShun MaekawaJ. S. Hill GastonShunbin NingJ. Stephen LodmellShuo ZhangJ.A.A.W. ElemansShuping TongJacek KuzmakShyam MoreJack H.Z. WuShyamtanu ChattorajJack StapletonSiamak ZohariJackie MaharSiddharth R. KrishnamurthyJacomine K. Krijnse-ĹockerSiddharth SridharJacquelina WoodsSiddhartha DattaJacqueline E. TateSigrid MillesJacqueline M. NoltingSilvia Ayora HirschJacques Davy IbabaSilvia CarnacciniJacques Le PenduSilvia FacciniJacques RobertSilvia García-BujalanceJacques TheronSilvia Gómez-SebastiánJade ForwoodSilvia GramolelliJae Myoung NohSilvia IppolitoJaeHun CheongSílvia MonteiroJae-Ku OemSilvia PreziusoJagdish KhubchandaniSilvia SauledaJai Singh PatelSimon D. Van HarenJaime Vera RojasSimon RouxJamel MankouriSimona AnticoliJames A. EllisonSimona KrabergerJames F. PapinSimona ZaamiJames F. StriebelSimone BaiardiJames FoxSimone BelliJames J. BullSimone BrogiJames KiruiSimone GiannecchiniJames LogueSimons SvirskisJames McNallySina BavariJames NaismithSindy BöttcherJames R. CarterSinu P. JohnJames S. (Jim) KoopmanSi-Qing LiuJames SchoelzSirle SaulJames SlukaSisko TauriainenJames Van EttenSivan LeviyangJames ZhuSlim FouratiJamie J. ArnoldSnjezana Zidovec LepejJan ClementSo NakagawaJan MarchantSofia CasaresJan PaczesnySoichiro TakahamaJan Pieter KonsmanSolomon MainaJan RybnikerSolomon O. OdemuyiwaJan WeberSomayeh PouyanfardJana L. JacobsSonia MorettiJana SchulzSonia SpandoleJana SmahelovaSonia ZuñigaJandouwe VillingerSonja BestJane OakeySonthaya TiawsirisupJanenuj WongtavatchaiSonu SubudhiJanet MansSoon Young PaikJanet NaleSophia ChouJanis MüllerSophie A. ValkenburgJansons JurisSophie ClementJaquelin P. DudleySophie Le PoderJared C RoachSoraya MezouarJared MaySouleymane DoucoureJasna Lenicek KrlezaSoumen BeraJason BodilySourish GhoshJason C. HuangSoyhan BagciJason E. ComerSpyridon MegremisJason E. ShoemakerSpyridon StavrouJason KaelberSreekumar OthumpangatJason L. LarabeeSridar ChitturJason McLellanSrihari KonduriJason ParisSrikanth ManamJason W. RauschSriramana KanginakudruJavier BuesaSt Patrick ReidJavier Castillo-OlivaresStanca CiupeJavier HerreroStavroula GiannouliJavier JusteStefan BauerJavier OrtegoStefan D. MomčilovićJay MelliesStefan HertwigJay R RadkeStefan LohseJayanta BhattacharyaStefan LüthJ.E. GumperzStefan RothenburgJean DubuissonStefan WegerJean Luc A.N. MurkStefan WorgallJean Luc E. DarlixStefania LeopardiJean Michel MesnardStefania Mirela MangJean Paul PirnayStefanie BarbirzJean Philippe KevorkianStefano AquaroJean-Michel LettStefano RealeJeanne Brugère-PicouxSteffan T. NawrockiJean-Philippe MarelliSteffen WagnerJean-Pierre FrossardStephan MenneJean-Pierre HernalsteensStephane BertagnoliJean-pierre MolesStephane LemiereJeeva SubbiahStéphane PrietJeff WiluszStephanie GamezJefferson SantosStephanie PfaenderJeffrey A. JohnsonStephanie PfänderJeffrey BaraStephanie SeifertJeffrey J. AdamoviczStephen BeebeJeffrey LotzStephen C. HardiesJemma L. GeogheganStephen CrookeJenna J GuthmillerStephen HsuJennifer AltomonteStephen HughesJennifer KopankeStephen JenningsJennifer L. CannonStephen KalesJennifer MytychStephen M. LaidlawJenq-Shyong ChanStephen M. RichJens André HammerlStephen MartinJens HahneStephen PolyakJens Von EinemStephen SpatzJeremy JonesSteve DunhamJeremy Russell KeownSteve WelchJeremy ThompsonSteven BradfuteJeremy V. CampSteven F. BakerJeremy W. ProkopSteven HallamJérôme BouchetSteven M. HeatonJerome E. TannerSteven M. PascalJerry W. SimeckaSteven M. ReedJesper Eugen-OlsenSteven M. SzczepanekJesse ArbuckleSteven PattersonJesse DeereSteven ReidJesse ErasmusSteven T PeperJessica BelserSteven VallesJessica CoertseStipan JonjicJessica J. HarrisonStivalis Cardenas-GarciaJessica J. O’KonekStuart T HamiltonJessica SmithStuart TurvilleJessie TrujilloSubir SarkerJesus HernandezSubramaniam SaravananJesus RequenaSubrata DebJesús Rodríguez-DíazSudarsana PoojariJevrosima StevanovicSukhmani BediJhon Castaño-OsorioSu-Mi KimJia LiuSumit MukherjeeJia Rong JhengSundaresh ShankarJian XieSunitha JosephJian YeSunitha KodidelaJianfeng LuSurendra KarkiJiang ZhongSuresh K. TikooJianmei W. LeavenworthSusan ChristoJianming TangSusan CorkJiann-Horng LeuSusan E. BuskinJiann-Ruey HongSusan L. FinkJianqiang ZhangSusan MooreJianxun QiSusan Nadin-DavisJie CuiSusan ShrinerJieshi YuSusan WilliamsJiezhong ChenSusana De Souza LalicJilei ZhangSusana GuixJilong ChenSusanna ChioccaJim M Pr DunwellSusanne G. DudmanJim StrongSuvi SutelaJimmy DikeakosSuzanne Faure-DupuyJin Il KimSven BergmannJin WeiSvetlana AntonyukJin-Der WenSwati JainJing JinSweilem B. Al RihaniJingjing TangSyed AhmedJingyun MaSyed Azmal AliJinhong ChangSylvain De BreyneJin-Won SongSylvia Franke McDevittJiri BeranSylvia RothenbergerJiří HejnarSylvie DallotJishu ShiSzecheng LoJisu LiSzu-Cheng KuoJitendra K. BiswalSzu-Wei HuangJitka ForstovaTaehwan OhJiuzhou SongTae-Jin ChoiJ.M. RijksTaejoong KimJoan BoyesTaek LeeJoan KyulaTahseen H NastiJoana DiasTai-long PanJoanna CobbinTaisuke HorimotoJoanna Maj-PaluchTakashi OnoderaJoanna MuchaTakashi SatoJoanna Sztuba-SolinskaTakayuki MiuraJoanne HewittTakemi OtsukiJoAnne OliverTakuma ShibataJoão Henrique Ghilardi LagoTakumi KoshibaJoao MamedeTalha Bin EmranJoão MesquitaTamaki OkabayashiJoaquin Manzo-MerinoTamas KovacsJocelyne PiretTanushree DangiJodi A. HaddenTao DengJody Hobson-PetersTatsuo KandaJoe BaumanTatsuo ShiodaJoe IcenogleTatsuo SuzutaniJoe MymrykTatyana IlyichevaJoel D. BainesTécher HervéJoel V. ChuaTetsuo KoshizukaJohan NordgrenTetsuya FuruyaJohanna LindhalTheresa Li-Yun ChangJohanne PoudrierThijs J.W. Van De LaarJohannes WittmannTimothy John MahonyJohn AtkinsTina O'GradyJohn BarnesTingting QinJohn CarpenterTobias KramerJohn CaseyTobias SchmidtJohn David BeckhamTodd G. SmithJohn FlanneryTohru SuzukiJohn Hoon RimTomáš MoravecJohn J SuschakTomofumi KurobeJohn Lok Man LawTomoichiro OkaJohn M. CoffinTomomasa MatsuyamaJohn M. NichollsToshio HattoriJohn MackenzieTrino Ascencio-IbanezJohn MatsoukasTsuguru HayashiJohn McBrideTsung-Hsien ChangJohn P AtkinsonTuli MukhopadhyayJohn T. BatesV. BalamuruganJohnatan DinmanVahid FarzanehJohnathan Cooper-KnockVan Doorslaer KoenraadJohnathan D. GuestVelmurugan BalaramanJohnny D. CallahanVeronika PiačkováJohn-Paul GonzalezVéronique DelesalleJohnson MakVicente MedinaJolanda J.C. VoermansVictoria MatyushenkoJolanda SmitVijay ReddyJolanta KiełpińskaVinay PathakJoLynn P. MontgomeryVincenza GianfrediJonathan AudetVincenzo SpagnuoloJonathan B. AngelViorica Railean-PlugaruJonathan BallVyacheslav MelnikovJonathan DordickWai-Lung NgJonathan GriffithsWang DanJonathan IpserWataru MitsuhashiJonathan LecureuxWathes D. ClaireJonathan O’Keeffe AhernWei CunJonathan P. LeisWeili KongJonathan Paul StoyeWeixin JiaJonathan R. BrestoffWen ChangJonathan RaynerWen SuJonathan StoyeWenliang LiJongseo MoWenping GongJong-Soo LeeWen-Sen LeeJong-Won OhWilliam KlitzJoon-seok ChaeWilliam N. BattsJordan ThomasWilliam R. RodríguezJordi Serra-CoboWilly M J M BogersJörg WennmannWin SurachetpongJorge C.G. BlancoXiaohong LiuJorge QuarleriXiaoling PanJorge Reyes-del ValleXiaowei GeJorma HinkulaXiaowen ChengJos M.B.M. Van Der VossenXiulian SunJosanne H. VerhagenXiuqiong BiJose A. Regla-NavaXun ChenJosé AlmendralYa-Chung TianJosé Avendaño-OrtizYanxin HuJose C. MartinezYasuhiro KanataniJosé ChiesYasutoshi KidoJosé González-SantamaríaYen-Hung ChowJosé Ignacio NúñezYifeng JiangJosé Luis AffranchinoYiming BaoJose Luiz ModenaYonggang LiJosé Manuel CabedaYonggang PeiJosé Sebastián DambolenaYonggyun KimJosep QuerYongjun SuiJoseph A. WestrichYoshihiro IkuraJoseph Calvin KouokamYoshihiro SakodaJoseph GloriosoYoshinori NakazawaJoseph HughesYouichi SuzukiJoseph MarcotrigianoYoukyung H. ChoiJoseph Michael Ochieng' OduorYoung Bong KimJoseph Thomas OrtegaYuan-Ti LeeJoshua SealyYuan-Yen ChangJózef J. BujarskiYu-Chan ChaoJuan Ángel Patiño-GalindoYuki FuruseJuan BarcenaYuko MorikawaJuan Bautista De SanctisYuriy DavidyukJuan C. de la TorreYuuki KurebayashiJuan C. HernandezZara SteinmeyerJuan Carlos Mora DíazZhong PengJuan Carlos ZapataZiwen WangJuan José BadiolaZoltán DeimJuan José López-MoyaZvi GrossmanJuan Manuel Pericas



